# Sports App Involvement and Core Literacy in Physical Education and Health Among University Students: Randomized Experiment and Cross-Sectional Study

**DOI:** 10.2196/104663

**Published:** 2026-11-11

**Authors:** Ruiyin Guo, Wencong Geng

**Affiliations:** 1 Department of Basic Courses, Qilu Institute of Technology, No. 3028 Jingshi East Road, Jinan, Shandong, 250200, China, 86 17861319670, 86 531 85592966; 2 School of Physical Education and Sport Science, Qufu Normal University, Qufu, Shandong, China; 3 School of Marxism (Public Teaching Department), Linyi City Vocational College, Linyi, Shandong, China

**Keywords:** sports app involvement, mobile health, physical activity, gamification, digitalization, core literacy in physical education and health

## Abstract

**Background:**

Sports apps can support physical activity through self-monitoring, feedback, and engagement features; however, evidence regarding their broader health-related outcomes remains mixed. The roles of digitalization and gamification in the association between sports app involvement and core literacy in physical education and health also remain unclear.

**Objective:**

This study aimed to evaluate a multicomponent app-supported physical activity intervention and examine the cross-sectional associations between sports app involvement and core literacy in physical education and health, including indirect statistical associations through digitalization and gamification.

**Methods:**

This study comprised 2 quantitative studies with independent samples. Study 1 used a Solomon 4-group randomized design in which 120 university students were allocated equally to the pretested experimental, pretested control, unpretested experimental, or unpretested control groups. The experimental groups received a 12-week multicomponent app-supported physical activity intervention, and 107 participants completed the postintervention assessment. A 2×2 factorial analysis of variance tested intervention and pretest main effects and their interaction, supplemented by a reference-based attrition sensitivity analysis. Study 2 surveyed 790 students from 8 universities and used latent-variable structural equation modeling to estimate direct and indirect cross-sectional associations, with age and sex included as covariates. The primary model assumed independence between participants; secondary composite-score sensitivity analyses examined additional covariate adjustment and university clustering.

**Results:**

In Study 1, significant intervention main effects were observed for self-reported motor competence (*F*_1,103_=33.869, *P*<.001, ηp²=0.247), health behaviors (*F*_1,103_=21.548, *P*<.001, ηp²=0.173), and sportsmanship (*F*_1,103_=30.261, *P*<.001, ηp²=0.227). Pretesting showed significant main effects on motor competence and health behaviors (both *P*<.001), but not on sportsmanship (*P*=.07). The pretest×intervention interactions were not statistically significant for motor competence (*P*=.92), health behaviors (*P*=.93), or sportsmanship (*P*=.79), and the attrition sensitivity analysis produced consistent results under the specified control-reference assumptions. In Study 2, sports app involvement was positively associated with core literacy (β=.135, *P*=.01). The largest specific indirect association was through digitalization (0.204, bias-corrected 95% CI 0.126‐0.290), followed by the sequential pathway through digitalization and gamification (0.036, bias-corrected 95% CI 0.005‐0.081) and gamification alone (0.031, bias-corrected 95% CI 0.004‐0.077).

**Conclusions:**

The multicomponent app-supported intervention yielded higher self-reported motor competence, health behavior, and sportsmanship scores than the control condition. In the independent cross-sectional sample, sports app involvement was positively associated with core literacy, with specific indirect associations through digitalization, gamification, and digitalization followed by gamification. Digitalization showed the largest specific indirect association. Further studies should assess objective outcomes and examine these associations longitudinally with broader measurement of potential confounders.

## Introduction

### Background

The integration of mobile health and wearable technologies into sports and exercise has enabled sports apps to support self-monitoring, feedback, and user engagement [1]. Reviews suggest that smartphone apps and activity trackers can increase physical activity, although benefits vary across interventions and outcomes [2-4]. Related research has examined physical activity, intervention engagement, continued app use, and behavioral intentions [5-9]. However, these outcomes do not fully capture broader competencies and dispositions, including motivation, confidence, physical competence, health-related decision-making, and sustained self-regulation [10-12].

In the Chinese educational context, core literacy in physical education and health is a policy-guided and theory-driven construct that reflects the fundamental competencies that individuals are expected to develop through physical education and health-related learning [13]. This concept emphasizes the integrated development of motor competence, health behaviors, and sportsmanship, highlighting an individual’s capacity to engage in physical activity in a sustained, responsible, and health-promoting manner. Although this construct originates from the Chinese educational system, it shares conceptual similarities with internationally discussed constructs such as physical and health literacy, while placing greater emphasis on integrating physical, behavioral, and value-oriented dimensions within physical activity contexts [10-13].

In this regard, previous studies have linked fitness app features to engagement and behavioral intentions and applied technology acceptance frameworks to wearable fitness technologies [8,9]. However, Conroy et al [14] found that behavior change techniques are underrepresented in top-ranked physical activity apps, suggesting that outcomes may depend on specific design features. Consequently, evidence regarding the effectiveness of sports apps remains unclear.

Digitalization of sports apps involves the quantification of activities through real-time monitoring and feedback. The technology acceptance model proposes that adoption is shaped by perceived usefulness and ease of use [15]. Digital monitoring converts physical activity into visible information such as steps, heart rate, and calories [16], while feedback can support progress monitoring, performance evaluation, and behavioral adjustment [17,18]. As these functions may strengthen perceived utility and support health-related self-regulation, digitalization represents a theoretically plausible app-related dimension associated with core literacy in physical education and health. Meanwhile, gamification applies game design elements such as leaderboards, badges, and challenges to nongame contexts [19,20]. Drawing on self-determination theory (SDT), such features may support autonomy, competence, and relatedness through choice, performance feedback, and social interaction [21,22]. Additionally, gamified challenges and achievement cues may encourage continued engagement [20,23,24] and support more autonomous motivation for sustained physical activity and health behavior regulation [25]. Furthermore, digitalization and gamification may be linked because gamified functions often rely on digitally recorded activity data [24].

### Objectives and Hypotheses

Therefore, consistent with calls to move beyond technology acceptance toward system features [26], this study examined digitalization and gamification as app-related dimensions associated with health-related outcomes using a 2-study quantitative design to provide complementary evidence on sports app–supported physical activity and core literacy in physical education and health. Study 2 examined a theory-guided cross-sectional association model linking sports app involvement, digitalization, gamification, and core literacy in physical education and health.

The following hypotheses were proposed for Study 1 and Study 2.

- Study 1
  - H1a: Students assigned to the multicomponent app-supported physical activity intervention group will demonstrate significantly higher postintervention levels of core literacy in physical education and health than those assigned to the control group.
  - H1b: Pretest exposure will not modify the intervention-control difference in postintervention core literacy scores.
- Study 2
  - H2: Sports app involvement is positively associated with core literacy in physical education and health.
  - H3: Sports app involvement has a significant positive indirect association with core literacy in physical education and health through digitalization.
  - H4: Involvement in sports apps has a significant positive indirect association with core literacy in physical education and health through gamification.
  - H5: Sports app involvement will show a significant sequential indirect association with core literacy in physical education and health through digitalization and gamification.

The specific hypothesized model diagram is shown in Figure 1.

**Figure 1.**
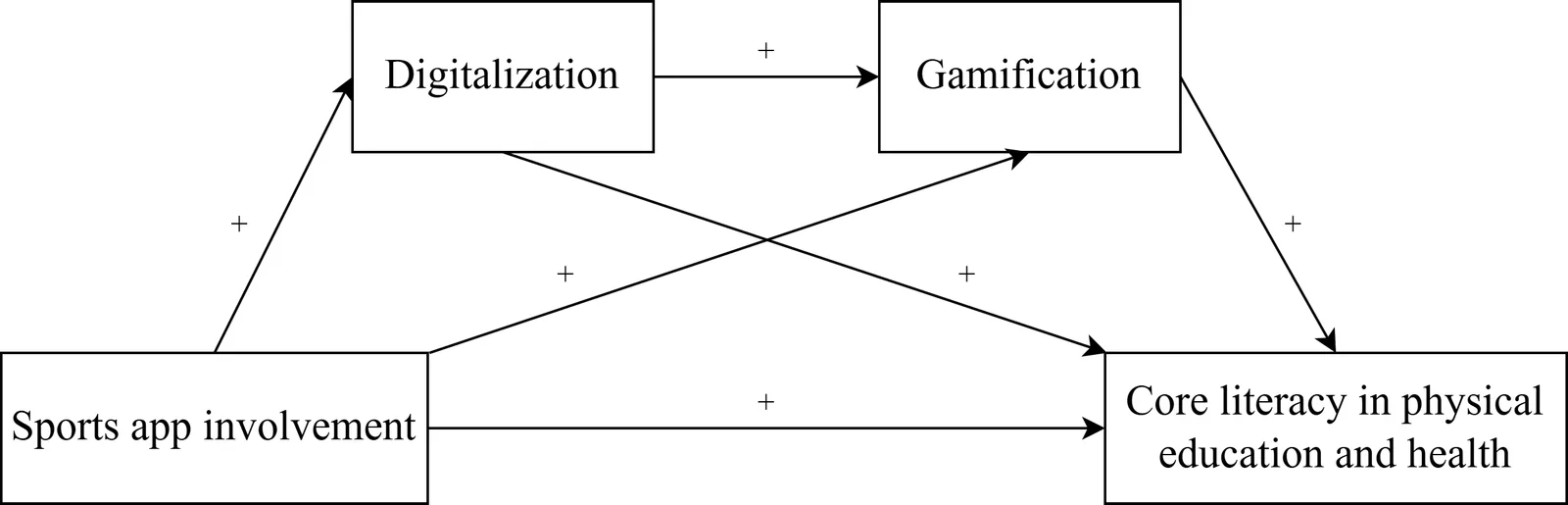
Hypothesized cross-sectional association model. Arrows represent theory-guided statistical pathways examined in Study 2 and do not establish temporal or causal ordering.

## Methods

### Ethical Considerations

The study protocol was reviewed and approved by the Biomedical Ethics Committee of Qufu Normal University (approval number 2023097). All participants were informed about the study purpose, procedures, potential risks and benefits, voluntary nature of participation, and their right to withdraw from the study at any time without penalty. Written informed consent was obtained from all participants prior to participation.

The randomized intervention component of this study was not prospectively registered before participant enrollment. The first participant was enrolled on June 27, 2023, and the intervention began on July 3, 2023. The trial was retrospectively registered in the Chinese Clinical Trial Registry (registration number: ChiCTR2600124527) on May 13, 2026. This delay occurred because the study was initially approved and implemented as a university-based educational and behavioral intervention. The retrospectively registered record is consistent with the ethics-approved protocol, and no post hoc changes were made to the intervention procedures, outcome measures, or main analysis plan. Participant data were deidentified before analysis, kept confidential, and used only for research purposes.

Participants in Study 1 received a commemorative gift valued at approximately RMB 100 (RMB 1=US $0.14 as of September 25, 2023) per participant as a token of appreciation for their participation. Participants in Study 2 received no compensation. No identifiable images of study participants are included in the manuscript or its supplementary materials.

### Study Design

#### Overview of the 2 Studies

This study used a 2-study quantitative design in which the 2 studies addressed complementary but distinct research questions. In Study 1, the theory of planned behavior (TPB) [27] and SDT [21] informed the multicomponent intervention. In educational and behavioral research contexts, baseline pretesting may influence participants’ subsequent attitudes or behaviors through familiarity, learning, or recall, thereby threatening internal validity [28]. This phenomenon is commonly referred to as the practice effect, or pretest sensitization. A Solomon 4-group randomized design was used to evaluate the effect of a 12-week multicomponent app-supported physical activity intervention on university students’ core literacy in physical education and health, while accounting for potential pretest sensitization. Because the intervention incorporated structured physical activity, app-based monitoring and feedback, health education, gamified activities, peer interaction, goal setting, reminders, and researcher support, the experimental contrast estimated the effect of the intervention package rather than the isolated effect of the sports app.

In Study 2, we used the technology acceptance model [15] and SDT [21], an independent cross-sectional survey sample, and structural equation modeling (SEM) to examine the direct and indirect associations among sports app involvement, digitalization, gamification, and core literacy in physical education and health. In the Study 2 model, digitalization and gamification were specified as functional and experiential app-related dimensions, respectively.

#### Study 1: Solomon 4-Group Design

##### Study Design and Sample Size

Given the 4-group Solomon design and anticipated 10% to 20% attrition, 120 participants were recruited, allowing 30 participants each to be assigned to the following groups: pretested experimental (PE), pretested control (PC), unpretested experimental (UE), and unpretested control (UC) groups. Among the included participants, 13 randomized participants did not complete the postintervention assessment. Therefore, the final sample with available postintervention outcome data comprised 107 participants: 28 in the PE group, 25 in the PC group, 25 in the UE group, and 29 in the UC group.

A schematic of the Solomon 4-group experiment is presented in Table 1. The completed CONSORT-EHEALTH (Consolidated Standards of Reporting Trials of Electronic and Mobile Health Applications and Online Telehealth) reporting checklist for Study 1 is provided in Checklist 1.

**Table 1.** Schematic diagram of the Solomon 4-group experimental design.

Group	Pretest score	Treatment	Posttest score
PE^a^	O1	X^b^	O2
PC^c^	O3	—^d^	O4
UE^e^	—^d^	X^b^	O5
UC^f^	—^d^	—^d^	O6

a PE: pretested experimental group.

b X indicates the multicomponent app-supported physical activity intervention.

c PC: pretested control group.

d — indicates that the corresponding assessment or intervention was not administered.

e UE: unpretested experimental group.

f UC: unpretested control group

##### Randomization and Allocation

After eligibility screening and written informed consent were obtained from all 120 participants, randomization was performed using a computer-generated random sequence in SPSS (IBM Corp). The participants were randomly assigned to the PE, PC, UE, and UC groups in a 1:1:1:1 ratio, with 30 participants initially allocated to each group. The principal researcher generated a random sequence and assigned groups. Separate allocation concealment procedures were not used.

##### Participants and Eligibility Criteria

The participants were full-time undergraduate students aged 18 to 25 years recruited in Shandong Province through campus-based and online recruitment. Online recruitment and eligibility prescreening began on June 16, 2023. Eligible participants completed an electronic informed consent form and entered the formal study process between June 27 and 28, 2023, and the intervention was initiated on July 3, 2023. Eligible participants were required to (1) have prior experience using mobile fitness apps, (2) have no professional athletic training background, and (3) safely engage in moderate-intensity physical activity. Students with self-reported medical contraindications to exercise, such as cardiovascular or musculoskeletal conditions, were excluded. The participants received a commemorative gift as a token of appreciation for their time and participation.

##### Interventions

###### Intervention Design and Delivery

The 12-week intervention comprised a multicomponent app-supported physical activity program informed by the TPB [27] and SDT [21]. TPB-guided components addressed attitudes, subjective norms, and perceived behavioral control, whereas SDT informed support for autonomy, competence, and relatedness. Participants used the Yodo Run app for activity tracking and were scheduled to complete 3 physical activity sessions per week (36 sessions over 12 wk), with approximately 30 minutes per session and a target rating of perceived exertion of 12 to 14. The activities included aerobic and strength exercises, gamified tasks, team-based activities, goal setting, exercise planning, and participant-selected activities. The intervention progressed from app familiarization and attitude-focused activities in weeks 1 to 4 to peer- and team-based exercise activities in weeks 5 to 8, and then to goal setting, exercise planning, and more participant-directed activity in weeks 9 to 12.

###### Intervention Content and Human Support

The weekly tasks were posted on WeChat. Participants submitted app-generated screenshots for each scheduled session, which were reviewed by 2 researchers. Missing submissions were followed up on the same day, and procedural or app-related assistance was provided when required. The overall intervention framework is shown in Figure 2, and the complete week-by-week schedule, including the TPB focus and intervention components, is provided in Multimedia Appendix 1 (Table S1).

**Figure 2.**
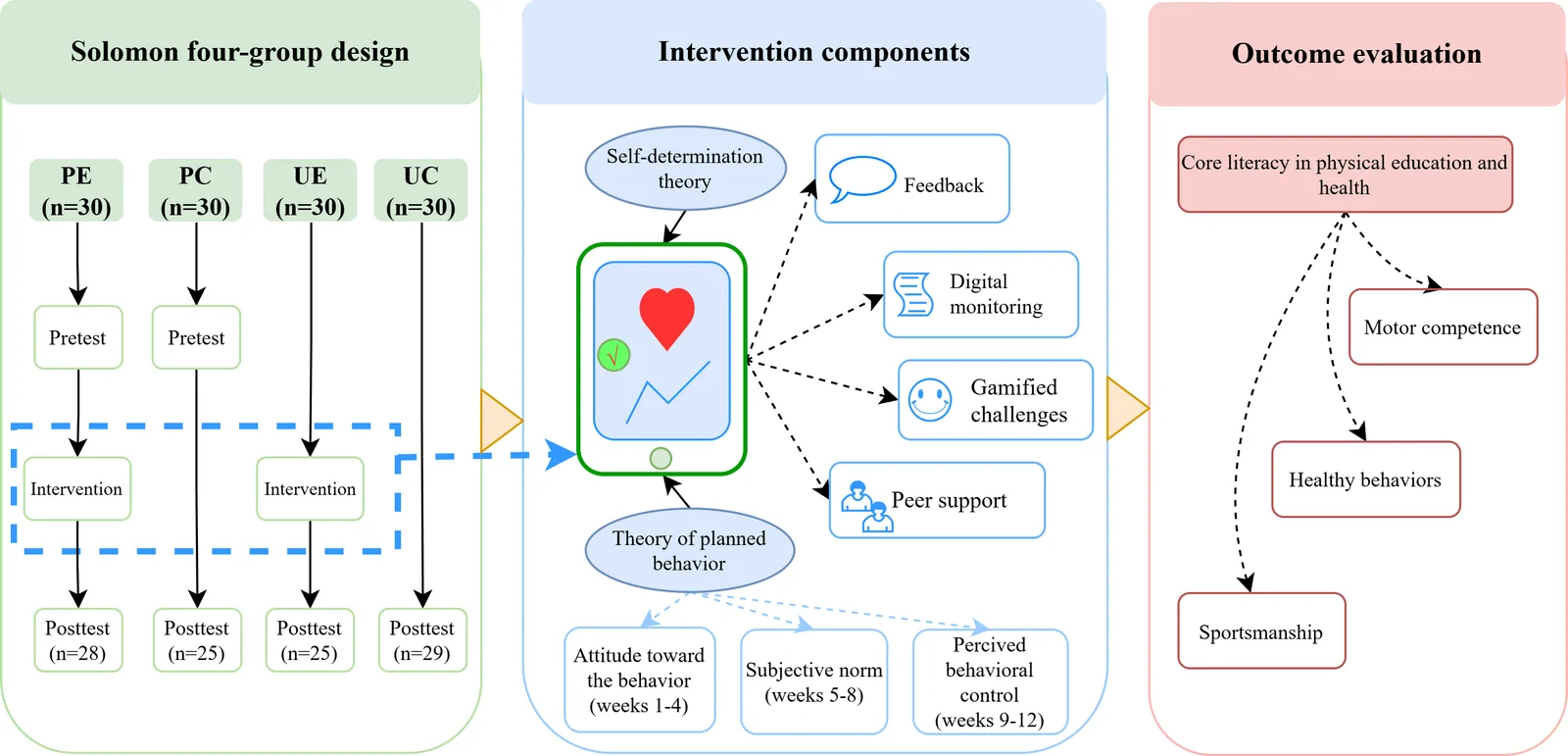
Solomon 4-group experimental design and intervention framework*.* PC: pretested control group; PE: pretested experimental group; UC: unpretested control group; UE: unpretested experimental group.

###### Control Condition and Adherence Monitoring

Participants in the control groups were included in separate WeChat groups, and routine activity records were submitted at the same frequency as the participants in the intervention groups. These records were used to document usual physical activity rather than adherence to a prescribed exercise intervention. The researchers checked the submitted records and provided same-day reminders when scheduled records were missing. The control group did not receive structured weekly exercise tasks, health education, gamified challenges, team-based intervention activities, exercise-planning tasks, or other behavior-change content provided to the intervention groups. Intervention adherence was monitored using the submitted app-generated records. A session was considered complete when the record documented approximately 30 minutes of the scheduled activity on the assigned day or within 24 hours. Participants who missed 3 or more scheduled sessions were classified as noncompliant for adherence monitoring.

###### Safety Monitoring

Exercise-related adverse events were monitored throughout the 12-week intervention period using participant reports and routine researcher contacts via WeChat.

### Study 2: Cross-Sectional Survey Design

Stratified sampling based on university hierarchy, primary specialty, and regional characteristics was used to invite 872 college students from 8 universities in Shandong Province to participate in a cross-sectional survey. Data were collected in November 2023. The participants in Study 2 were independent of those in Study 1. The surveys were conducted online and offline. After logical screening to exclude invalid responses, such as questionnaires completed in less than 120 seconds or containing extreme response patterns, 790 of the 872 (90.6%) questionnaires were retained. A root mean square error of approximation (RMSEA)-based power analysis was used to estimate the sample size required for testing the overall fit of the 11-indicator measurement model [29]. With 38 degrees of freedom, α=.05, a null RMSEA of 0.05, and an alternative RMSEA of 0.08, a sample of 262 would provide 80% power. The final sample size of 790 participants exceeded this benchmark.

### Measures

#### Questionnaire Development and Pilot Validation

The questionnaire drew on published instruments and relevant literature and was contextualized for sports app users and university students. Wording and contextual relevance were reviewed by 5 physical education experts, 3 doctoral students, and 8 master’s students, followed by student tryouts and refinements. Items were rated on a 5-point Likert-type scale, with higher scores indicating higher levels of the corresponding construct.

Before the formal Study 2 survey, the 4 instruments were evaluated using a pilot sample of 192 university students. Item screening was performed using the full pilot sample, after which the responses were divided into 2 subsamples comprising 96 university students each. One subsample was used for exploratory factor analysis of the app-related scales, and the other subsample was used for confirmatory factor analysis (CFA) of the retained measurement structures. The prespecified 3-domain core literacy structure was evaluated directly by CFA. Structural hypothesis testing used the subsequent independent 790-participant formal Study 2 sample. The detailed factor loadings, composite reliability (CR), and average variance extracted (AVE) are reported in Multimedia Appendix 1 (Table S2).

#### Sports App Involvement Scale

The Sports App Involvement Scale was adapted from the leisure involvement framework of McIntyre and Pigram [30] and contextualized for sports app use. The 10-item instrument retained 3 dimensions: attractiveness (4 items), self-expression (3 items), and life centrality (3 items). In the pilot validation, the 3-factor measurement model showed acceptable fit (χ²/*df*=1.479, standardized root mean square residual [SRMR]=0.038, RMSEA=0.071, and comparative fit index [CFI]=0.968).

#### Digitalization Scale

Digitalization was assessed using an 8-item scale adapted from the monitoring and feedback measures reported by Nelson et al [18], with item wording contextualized from activity trackers to sports apps. The monitoring dimension comprises 4 items addressing the amount, presentation, constructiveness, and frequency of information, whereas the feedback dimension comprises 4 items addressing perceived accuracy, usefulness, effectiveness, and reliability. In the pilot validation, the 2-factor measurement model showed acceptable fit (χ²/*df*=1.365, SRMR=0.038, RMSEA=0.062, and CFI=0.977).

#### Gamification Scale

The Gamification Scale was developed for the sports app context, rather than being directly adopted from a single existing instrument. The dimensional structure of this scale was informed by the SDT and prior research on motivational and game-related experiences [21,22]. Following literature review and expert refinement, gamification was operationalized using 9 items across 3 dimensions: sense of mastery, interaction fluency, and intrinsic motivation. In the pilot validation, the 3-factor measurement model showed acceptable fit (χ²/*df*=1.228, SRMR=0.042, RMSEA=0.049, and CFI=0.979).

#### Physical Education and Health Core Literacy Scale

The Physical Education and Health Core Literacy Scale was developed for this study based on the 3-domain framework specified in the Chinese Physical Education and Health Curriculum Standards and informed by earlier Chinese instruments assessing core literacy among university students. The initial scale included 30 items covering motor competence, health behaviors, and sportsmanship. However, items D9, D14, D29, and D30 were removed during the pilot item analysis. Items D9, D14, and D29 did not meet the prespecified extreme-group discrimination criterion (*t*≥3.00), whereas item D30 had a corrected item-total correlation of 0.374, which was below the 0.400 criterion. The resulting 26-item scale comprised 8 motor competence items, 11 health behavior items, and 7 sportsmanship items and was used in the formal survey. Item removal was guided by prespecified psychometric criteria while retaining the representation of all 3 theoretically defined domains, thereby preserving the intended 3-domain structure of the scale. The retained 3-factor measurement model showed acceptable fit in the pilot CFA (χ²/*df*=1.267, SRMR=0.045, RMSEA=0.053, and CFI=0.948).

In both studies, core literacy in physical education and health and its 3 dimensions were assessed using participant-reported questionnaires. The sources, adaptation procedures, and final structures of the 4 Study 2 instruments are summarized in Multimedia Appendix 1 (Table S3).

### Data Analysis

#### Study 1

Study 1 analyses were conducted using SPSS (version 27.0). The primary analysis was a 2×2 factorial ANOVA of postintervention scores, with pretest exposure and intervention condition as between-participant factors. As a supporting analysis, postintervention scores in the pretested experimental and control groups were compared using analysis of covariance (ANCOVA), adjusting for the corresponding baseline scores. Group-by-baseline interaction terms were examined to assess the homogeneity of regression slopes. These analyses included participants with available postintervention outcomes. Baseline characteristics of completers and noncompleters in the pretested groups were compared using Welch independent-samples *t* tests and Fisher exact tests, as appropriate.

Sensitivity to the 13 missing postintervention assessments was examined using reference-based resampling with 10,000 replications. Within each pretest stratum, missing outcomes in the intervention groups were sampled with replacement from the observed outcome vectors in the corresponding control group, assuming no intervention benefit among intervention-group noncompleters. Missing control-group outcomes were sampled from the corresponding observed control-group outcomes. The 3 outcomes were sampled jointly to preserve their within-participant correlation structure. The structural absence of pretest measurements in the unpretested groups was not treated as missing data. This sensitivity analysis supplemented the complete-case analyses; it did not constitute a conventional intention-to-treat analysis.

#### Study 2

Study 2 analyses used SPSS (version 27.0) and Amos (version 29.0). Internal consistency was assessed using Cronbach α and discriminant validity using the heterotrait-monotrait ratio. Common method effects were examined using Harman’s single-factor test and a CFA comparison of the hypothesized 4-factor and 1-factor models. Model fit was evaluated using χ²/*df*, CFI, Tucker-Lewis index (TLI), RMSEA, and SRMR. Descriptive statistics and Pearson correlations were also calculated.

The primary age- and sex-adjusted latent-variable SEM estimated direct and indirect associations, with bias-corrected (BC) CIs obtained from 5000 bootstrap resamples. The latent-variable specification was retained to account for measurement error in the construct indicators. The inclusion of only 8 universities provided limited university-level information for a full multilevel latent model. The primary SEM assumed independence between participants, and its SEs and bootstrap CIs were not adjusted for university-level clustering.

University-level clustering was described using intercept-only random-effects models, intraclass correlation coefficients, and design effects. Two secondary analyses used standardized composite scores. One added BMI and 7 university indicator variables to the age- and sex-adjusted models. The other applied university-clustered robust standard errors to the age- and sex-adjusted composite-score models, with a finite-sample correction and t-based inference using 7 *df*, implemented in Python (version 3.13.5) with statsmodels (version 0.14.6). These analyses assessed the consistency of the 6 composite-score paths; cluster-adjusted CIs for the primary latent-model indirect associations were not estimated.

## Results

### Study 1: Randomized Experiment Results

#### Participant Flow and Attrition

Of the 120 randomized participants, 107 (89.2%) completed the postintervention assessment: 93% (28/30) in the PE group, 83% (25/30) in the PC group, 83% (25/30) in the UE group, and 97% (29/30) in the UC group. In total, 45.8% (49/107) of the participants who completed the postintervention assessment were men; the complete-case sample had a mean age of 20.56 (SD 1.59) years. Detailed participant characteristics are provided in Multimedia Appendix 1, Table S4, and the reasons for 10.8% (13/120) noncompletions are shown in Figure 3.

Among the 60 pretested participants, 53 completed the postintervention assessment and 7 did not. Completers and noncompleters did not differ significantly at baseline in sex, age, anthropometric characteristics, or core literacy measures (Multimedia Appendix 1, Table S5). No exercise-related adverse events were reported.

**Figure 3.**
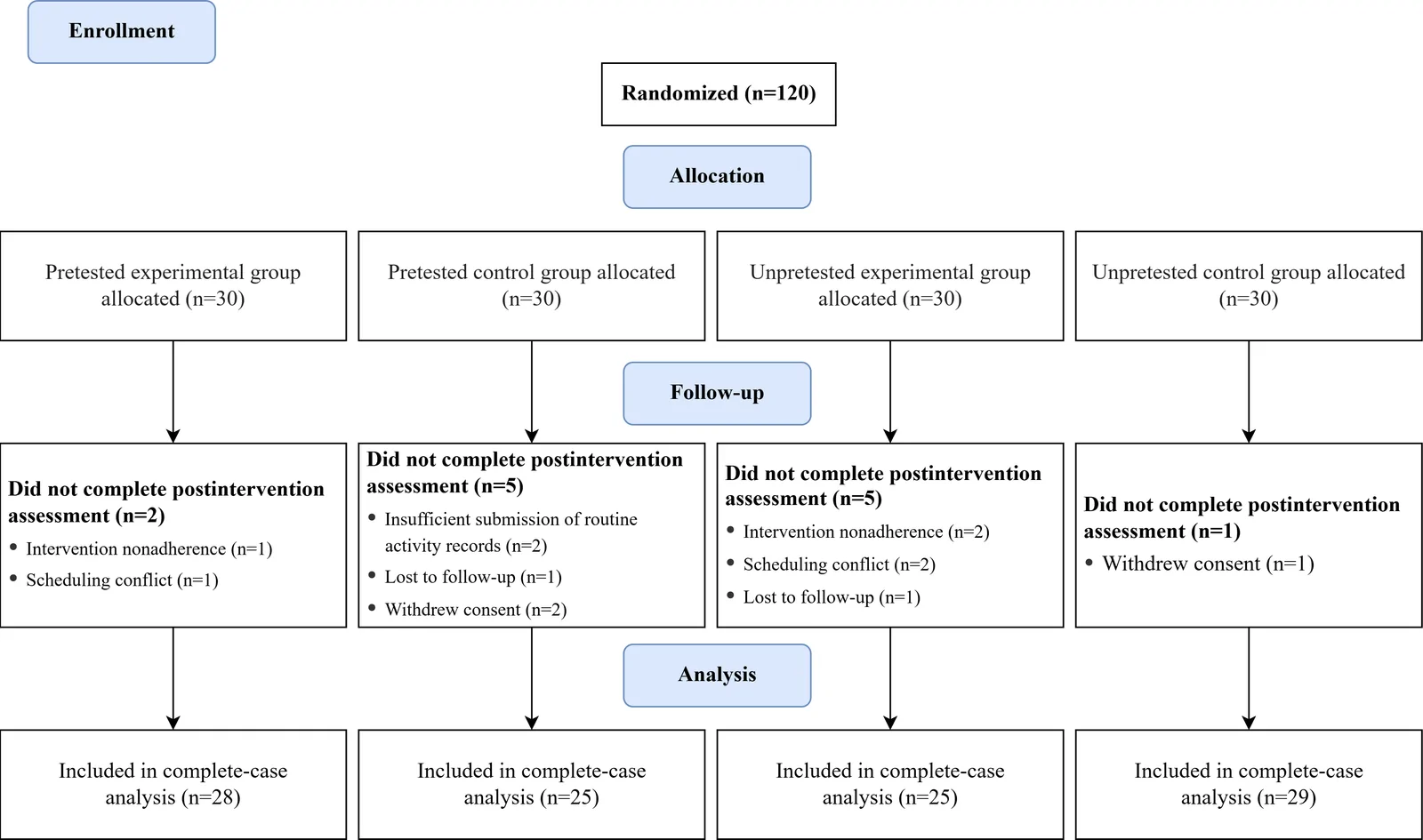
Participant flow through Study 1.

#### Primary Factorial Analysis of Intervention and Pretest Effects

The primary analysis in Study 1 was a 2×2 factorial ANOVA of posttest scores, with pretest exposure and intervention conditions as the between-group factors. Significant intervention main effects were observed for self-reported motor competence (*F*_1,103_=33.869, *P*<.001, ηp²=0.247), health behaviors (*F*_1,103_=21.548, *P*<.001, ηp²=0.173), and sportsmanship (*F*_1,103_=30.261, *P*<.001, ηp²=0.227), with higher scores in the intervention condition (Table 2).

**Table 2.** Summary of 2×2 factorial ANOVA with posttest scores as dependent variables.

Outcome and source	Type III SS^a^	MS^b^	*F* test (*df*)	*P* value	ηp^2^^c^
Motor competence					
Pretest (A)	284.170	284.170	22.290 (1, 103)	<.001	0.178
Intervention (B)	431.783	431.783	33.869 (1, 103)	<.001	0.247
A×B	0.144	0.144	0.011 (1, 103)	.92	0.000
Error	1313.119	12.749	—^d^	—	—
Health behaviors					
Pretest (A)	401.367	401.367	19.489 (1, 103)	<.001	0.159
Intervention (B)	443.787	443.787	21.548 (1, 103)	<.001	0.173
A×B	0.161	0.161	0.008 (1, 103)	.93	0.000
Error	2121.284	20.595	—	—	—
Sportsmanship					
Pretest (A)	22.702	22.702	3.478 (1, 103)	.07	0.033
Intervention (B)	197.507	197.507	30.261 (1, 103)	<.001	0.227
A×B	0.473	0.473	0.072 (1, 103)	.79	0.001
Error	672.260	6.527	—	—	—

a SS: sum of squares.

b MS: mean square.

c ηp²: partial eta squared.

d Not applicable.

Pretesting showed significant main effects on motor competence (*F*_1,103_=22.290, *P*<.001, ηp²=0.178) and health behaviors (*F*_1,103_=19.489, *P*<.001, ηp²=0.159), but not on sportsmanship (*F*_1,103_=3.478, *P*=.07, ηp²=0.033). The Pretest×Intervention interaction was not statistically significant for motor competence (*F*_1,103_=0.011, *P*=.92, ηp²=0.000), health behaviors (*F*_1,103_=0.008, *P*=.93, ηp²=0.000), or sportsmanship (*F*_1,103_=0.072, *P*=.79, ηp²=0.001), providing no evidence that the intervention effect differed according to pretest exposure.

#### Supporting ANCOVA in the Pretested Groups

As a supporting analysis among the pretest groups, posttest outcomes were compared between the PE and PC groups using an ANCOVA while controlling for baseline scores. The group-by-baseline interaction terms were not statistically significant across the three outcomes, supporting the assumption of homogeneity in the regression slopes.

After adjustment for baseline scores, the intervention group had higher self-reported posttest scores than the control group for motor competence (*F*_1,50_=12.703, *P*<.001, ηp²=0.203), health behaviors (*F*_1,50_=9.481, *P*=.003, ηp²=0.159), and sportsmanship (*F*_1,50_=14.713, *P*<.001, ηp²=0.227). These results were consistent with those of the primary factorial analysis (Table 3).

**Table 3.** Summary of analysis of covariance for the pretested experimental and control groups.

Outcome and source	Type III SS^a^	MS^b^	*F* test (*df*)	*P* value	ηp^2^^c^
Motor competence					
Pretest	47.449	47.449	3.565 (1, 50)	.06	0.067
Intervention	169.091	169.091	12.703 (1, 50)	<.001	0.203
Error	665.551	13.311	—^d^	—	—
Health behaviors					
Pretest	22.065	22.065	1.039 (1, 50)	.31	0.020
Intervention	201.343	201.343	9.481 (1, 50)	.003	0.159
Error	1061.859	21.237	—	—	—
Sportsmanship					
Pretest	2.048	2.048	0.343 (1, 50)	.56	0.007
Intervention	87.855	87.855	14.713 (1, 50)	<.001	0.227
Error	298.562	5.971	—	—	—

a SS: sum of squares.

b MS: mean square.

c ηp²: partial eta squared.

d Not applicable.

#### Attrition Sensitivity Analysis

Under the specified control-reference assumptions, the reference-based resampling sensitivity analysis yielded results consistent with those of the complete-case analysis. Across all 10,000 replicates, the main effect of the intervention remained statistically significant for motor competence, health behaviors, and sportsmanship. The median intervention-effect estimates for motor competence, health behaviors, and sportsmanship were *F*_1,116_=27.60 (ηp²=0.192), *F*_1,116_=17.94 (ηp²=0.134), and *F*_1,116_=24.52 (ηp²=0.175), respectively. The detailed simulation summaries are provided in Multimedia Appendix 1 (Table S6).

#### Exploratory Post Hoc Comparisons

Detailed exploratory Tukey honestly significant difference comparisons are provided in Multimedia Appendix 1 (Table S7). In brief, the corresponding intervention-control contrasts were statistically significant for all 3 outcomes under the pretested and unpretested conditions; exact *P* values are reported in Table S7 in Multimedia Appendix 1. However, as the pretest × intervention interactions were not statistically significant, these pairwise contrasts were interpreted as descriptive rather than primary evidence.

### Study 2: Cross-Sectional Survey Results

#### Participant Characteristics and University Clustering

The final Study 2 sample comprised 790 university students, of whom 409 (51.8%) were men. The mean age of this sample was 20.33 (SD 1.78) years, with an age range of 18 to 25 years. Detailed demographic characteristics and university-specific sample sizes ranging from 61 to 117 per university are presented in Multimedia Appendix 1 (Table S8). The intraclass correlation coefficients for sports app involvement and digitalization were 0.010 and 0.002, respectively, whereas those for gamification and core literacy in physical education and health were approximately 0; the corresponding design effects were 1.99, 1.18, 1.00, and 1.00, respectively (Multimedia Appendix 1, Table S9).

#### Measurement Reliability, Convergent Validity, and Discriminant Validity

In the pilot CFA subsample (n=96), the standardized factor loadings for the retained items ranged from 0.604 to 0.834. AVE values across the 11 first-order dimensions ranged from 0.508 to 0.617, and CR values ranged from 0.757 to 0.934, supporting convergent validity (Multimedia Appendix 1, Table S2).

The final Study 2 sample had good internal consistency, with Cronbach α values of 0.890 for sports app involvement, 0.848 for digitalization, 0.814 for gamification, and 0.924 for core literacy in physical education and health. In the formal 4-factor measurement model, standardized dimension-level factor loadings ranged from 0.703 to 0.906, CR values ranged from 0.770 to 0.865, and AVE values ranged from 0.527 to 0.683. The heterotrait-monotrait ratios among the four focal constructs ranged from 0.426 to 0.638, with all values below the conservative 0.85 criterion, supporting the empirical distinction among the four constructs (Multimedia Appendix 1, Tables S10 and S11).

#### Assessment of Common Method Bias

Harman single-factor test identified nine factors with eigenvalues >1, with the first factor accounting for 25.1% of the total variance. The hypothesized 4-factor model showed an acceptable fit (χ²_38_=129.39, χ²/*df*=3.405, CFI=0.978, TLI=0.968, RMSEA=0.055, SRMR=0.030), whereas the 1-factor model showed a poor fit (χ²_44_=1401.80, χ²/*df*=31.859, CFI=0.670, TLI=0.587, RMSEA=0.198, SRMR=0.119). These findings argue against a single dominant common factor; however, common method effects cannot be ruled out because the focal measures were self-reported and collected simultaneously.

#### Correlation Analysis

Descriptive statistics and correlations are presented in Table 4. Correlations among the 4 focal constructs were positive, ranging from *r*=0.386 to *r*=0.538 (all *P*<.001).

**Table 4.** Descriptive statistics and Pearson correlations for Study 2 variables.

Variables	Mean (SD)	1	2	3
Sports app involvement	35.123 (6.831)	—^a^	—	—
Digitalization	30.565 (4.606)	0.538	—	—
Gamification	34.527 (5.085)	0.477	0.529	—
Physical education and health core literacy	103.235 (13.842)	0.388	0.462	0.386

a Not applicable.

#### Structural Model

The primary single-level latent-variable model showed an acceptable fit for the cross-sectional data (χ²_54_=185.892, χ²/*df*=3.442, CFI=0.968, TLI=0.953, RMSEA=0.056, 90% CI 0.047‐0.064). Sports app involvement was associated with core literacy in physical education and health (β=.135, *P*=.01), digitalization (β=.653, *P*<.001), and gamification (β=.279, *P*<.001). Digitalization was associated with core literacy (β=.374, *P*<.001) and gamification (β=.486, *P*<.001), and gamification was associated with core literacy (β=.135, *P*=.02; Table 5 and Figure 4).

Inference from this primary model was based on the assumption of independence between participants.

The primary latent model was adjusted for age and sex and assumed independence between participants; its SEs were not adjusted for university-level clustering.

**Table 5.** Structural path coefficients of the primary latent-variable structural equation model.

Path	B^a^ (SE)	*z* ^b^	*P* value	β^c^
App involvement → Core literacy	0.113 (0.046)	2.470	.01	.135
App involvement → Digitalization	0.578 (0.035)	16.372	<.001	.653
Digitalization → Core literacy	0.353 (0.061)	5.782	<.001	.374
App involvement → Gamification	0.217 (0.043)	5.002	<.001	.279
Gamification → Core literacy	0.145 (0.064)	2.256	.02	.135
Digitalization → Gamification	0.427 (0.053)	7.994	<.001	.486

a B: the unstandardized regression coefficient.

b z: critical ratio.

c β: the standardized regression coefficient.

**Figure 4.**
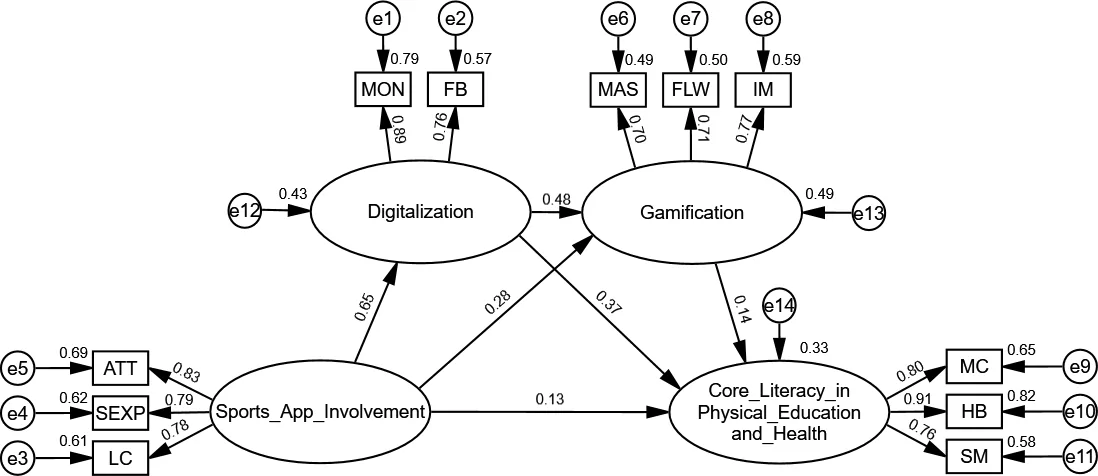
Latent-variable model of the cross-sectional associations among sports app involvement, digitalization, gamification, and core literacy in physical education and health. Values on paths between latent constructs are standardized regression coefficients; values on paths from latent constructs to their indicators are standardized factor loadings. Circles labeled e1 to e14 represent measurement errors or structural disturbances. Arrows represent statistical associations and do not establish temporal or causal ordering. ATT: attractiveness; FB: feedback; FLW: interaction fluency; HB: health behavior; IM: intrinsic motivation; LC: life centrality; MAS: sense of mastery; MC: motor competence; MON: monitoring; SEXP: self-expression; SM: sportsmanship.

#### Indirect Associations

Bootstrap analysis showed a model-estimated total association of 0.385 (BC 95% CI 0.296‐0.478). The direct association was 0.113 (BC 95% CI 0.016‐0.210), accounting for 29.5% of the model-estimated total association, and the total indirect association was 0.271 (BC 95% CI 0.194‐0.361), accounting for 70.5%. The specific indirect associations through digitalization (0.204, BC 95% CI 0.126‐0.290), gamification (0.031, BC 95% CI 0.004‐0.077), and digitalization followed by gamification (0.036, BC 95% CI 0.005‐0.081) accounted for 53.1%, 8.2%, and 9.3% of the model-estimated total association, respectively (Table 6).

**Table 6.** Model-estimated direct, indirect, and sequential indirect associations between sports app involvement and core literacy in physical education and health^a^.

Association	Estimate (BC 95% CI^b^)	Proportion of model-estimated total association (%)
Total association	0.385 (0.296-0.478)	100
Direct association	0.113 (0.016-0.210)	29.5
Total indirect association	0.271 (0.194-0.361)	70.5
Sports app involvement → digitalization → core literacy in physical education and health	0.204 (0.126-0.290)	53.1
Sports app involvement → gamification → core literacy in physical education and health	0.031 (0.004-0.077)	8.2
Sports app involvement → digitalization → gamification → core literacy in physical education and health	0.036 (0.005-0.081)	9.3

a Estimates are unstandardized cross-sectional associations adjusted for age and sex.

b BC 95% CI: bias-corrected 95% CI based on 5000 bootstrap resamples. CIs were obtained from the primary latent model without adjustment for university-level clustering.

#### Sensitivity Analysis

In the secondary sensitivity path analysis based on standardized composite scores, adding BMI and 7 university indicator variables to the age- and sex-adjusted models resulted in only small changes in the 6 focal coefficients (absolute changes ≤0.010), and all 6 focal paths remained statistically significant (all *P*<.001; Multimedia Appendix 1, Table S9, panel B). In the university-clustered analysis of the age- and sex-adjusted composite-score models, all 6 focal paths remained positive and statistically significant (all *P*<.05; Multimedia Appendix 1, Table S9, panel C). These secondary results supported the consistency of the 6 composite-score paths.

## Discussion

### Principal Findings

Study 1 showed higher participant-reported core literacy scores after the 12-week multicomponent app-supported intervention than under the control condition. In Study 2, sports app involvement, digitalization, gamification, and core literacy showed positive cross-sectional associations, with digitalization showing the largest model-estimated indirect association. These associations warrant further investigation in longitudinal and experimental studies.

### App-Supported Intervention and Core Literacy

Study 1 evaluated a multicomponent intervention package combining structured physical activity, app-based monitoring and feedback, health education, goal-setting, peer interaction, gamified challenges, reminders, and researcher support. Recent studies on digital interventions have suggested their selective rather than uniform benefits. A 2024 review of healthy young adults reported limited and inconsistent effects [31], whereas a 2024 JMIR meta-analysis of college students found increased step counts but no significant improvements in light or moderate-to-vigorous physical activity or sedentary time [32]. A 2025 meta-analysis of standalone digital behavior change interventions found modest improvements in physical activity, although the certainty of evidence for physical activity was low [33]. More recently, a 2026 systematic review of digital health interventions among college students reported generally favorable effects on physical activity while emphasizing limited evidence regarding long-term effectiveness [34]. Taken together, these findings suggest that digital interventions can support selected behavioral outcomes, but their effects vary according to the intervention design, population, outcome, and follow-up period.

### Testing the Effect of the Pretest

Pretesting showed significant main effects on self-reported motor competence and health behavior scores. Repeated exposure to assessment items may increase familiarity with the questionnaire and prompt reflection on exercise behaviors [28]. The Solomon 4-group design allowed the main effects of pretesting and intervention to be examined separately. The pretest×intervention interactions were not statistically significant for any of the 3 outcomes.

No statistically significant pretest main effect was detected for sportsmanship. Its value-oriented aspects develop gradually through sustained sports participation and social experience [35]. These relatively stable value orientations may be less responsive to a single exposure to assessment items.

### Digitalization, Gamification, and Their Indirect Associations

In the cross-sectional model in Study 2, digitalization showed the largest specific indirect association between sports app involvement and core literacy, accounting for 53.1% of the total model-estimated association. From a functional perspective, monitoring and feedback may help users recognize exercise progress, evaluate goal attainment, and adjust exercise plans, providing informational support for physical activity and health self-management. This interpretation aligns with a 2024 meta-analysis showing that self-monitoring interventions with feedback achieved better physical activity outcomes than those without feedback (Cohen *d*=0.29, 95% CI 0.16‐0.43) [36].

Positive indirect associations were also observed through gamification alone and through the model-estimated sequential pathway involving digitalization and gamification. Gamification may support enjoyment and motivation for participation, complementing the informational support provided by monitoring and feedback [37]. A 2024 meta-analysis reported a small increase in daily steps with gamified compared with nongamified apps [38], while a 2025 meta-analysis in individuals with cardiovascular disease identified feedback as the most important game design element in its meta-regression analysis [39]. From this perspective, feedback may provide a connection between digital functions and gamified experiences, offering a functional interpretation of the sequential association observed in this study. Longitudinal and experimental studies could further examine the integration of these functions and their relationship with changes in core literacy.

### Practical Implications

For university physical education, the findings support further evaluation of sports apps as tools within structured physical activity programs. Such programs may combine clear activity feedback and progress monitoring with attainable goals, participant choices, peer interactions, and selectively used gamified challenges. Evaluation should include objectively measured physical activity and performance, adherence, and long-term outcomes alongside self-reported competencies.

### Research Limitations and Future Directions

This study has several limitations. First, both studies involved university students, and Study 2 was limited to 1 province, restricting generalizability. The primary latent-variable SEM assumed participant-level independence; its SEs and CIs, including those for indirect associations, were not adjusted for university clustering and may understate uncertainty. Clustering was examined only in secondary composite-score models of the 6 focal paths, with inference limited by the small number of universities (n=8).

Second, Study 1 evaluated a multicomponent app-supported intervention, so the contributions of individual components could not be isolated. The intervention groups also received more structured content and task-related support than the control groups. The absence of allocation concealment posed a risk of selection bias.

Third, the cross-sectional design of Study 2 did not establish temporal ordering, and reverse or reciprocal relationships remain possible. Covariate adjustment did not address unmeasured usual physical activity, health status, or app use patterns, leaving potential residual confounding.

Fourth, 10.8% (13/120) of randomized participants did not complete the postintervention assessment, creating potential attrition bias in the complete-case Study 1 analyses. The reference-based sensitivity analysis yielded consistent findings under its specified missing-outcome assumptions. Retrospective trial registration also limited prospective verification of planned outcomes and analyses.

Finally, the pilot CFA subsample was not fully independent of item refinement because item screening preceded sample splitting. Structural hypotheses were tested in a separate formal sample. Self-reported outcomes may have been affected by social desirability and residual common method bias; objective physical performance and physical activity were not assessed. Future studies should prioritize multisite longitudinal or experimental designs, attention-matched active controls, independent measurement validation, and objective or multi-informant outcomes.

### Conclusions

Study 1 showed higher self-reported motor competence, health behavior, and sportsmanship scores among university students receiving the 12-week multicomponent app-supported physical activity intervention than among controls. The Solomon 4-group design identified pretest effects on motor competence and health behaviors, with no statistically significant pretest-by-intervention interaction for any of the 3 outcomes. In the independent Study 2 cross-sectional sample, sports app involvement was positively associated with core literacy, and digitalization showed the largest specific indirect association, followed by the sequential pathway through digitalization and gamification and the specific pathway through gamification alone. The findings support further evaluation of app-supported university physical activity programs with objective outcomes and attention-matched controls, alongside longitudinal testing of the Study 2 associations.

## Supplementary material

10.2196/104663 Multimedia Appendix 1 This appendix presents the intervention schedule, participant characteristics, measurement validation, attrition analyses, exploratory comparisons, and clustering and sensitivity analyses for Studies 1 and 2.

10.2196/104663 Checklist 1 CONSORT-EHEALTH checklist (V 1.6.1).

## Abbreviations

ANCOVA: analysis of covariance
AVE: average variance extracted
BC: bias-corrected
CFA: confirmatory factor analysis
CFI: comparative fit index
CONSORT-EHEALTH: Consolidated Standards of Reporting Trials of Electronic and Mobile Health Applications and Online Telehealth
CR: composite reliability
PC: pretested control group
PE: pretested experimental group
RMSEA: root mean square error of approximation
SDT: self-determination theory
SEM: structural equation modeling
SRMR: standardized root mean square residual
TLI: Tucker-Lewis index
TPB: theory of planned behavior
UC: unpretested control group
UE: unpretested experimental group
