# Supplementary material for: Sports App Involvement and Core Literacy in Physical Education and Health Among University Students: Randomized Experiment and Cross-Sectional Study
Source: JMIR Serious Games. 2026 Nov 11;14:e104663. doi: 10.2196/104663 (PMC13754180; doi:10.2196/104663)
Supplement: Supplementary file 2 [file games-v14-e104663-s001.docx]

Multimedia Appendix 1

Supplementary methods and tables for the attrition sensitivity analysis, baseline comparison between completers and noncompleters, the 12-week intervention schedule, participant characteristics, measurement validity, instrument adaptation, university-level clustering, covariate sensitivity analysis, and detailed exploratory Tukey HSD comparisons.

Table S1. Twelve-Week Intervention Schedule for Study 1

| Week | TPB focus | Weekly intervention task | Main component |
| --- | --- | --- | --- |
| 1 | Attitude toward the behavior | Introduction to the functions, characteristics, and potential benefits of the sports app | App orientation and perceived usefulness |
| 2 | Attitude toward the behavior | App installation/registration and guidance on basic app operation | App-use skills |
| 3 | Attitude toward the behavior | Participation in app-based sports games and challenges | Gamified exercise experience |
| 4 | Attitude toward the behavior | Exploration of health information and sports-related content through the app | Health information and app exploration |
| 5 | Subjective norms | App-supported learning and practice of warm-up and cool-down activities | Exercise learning and peer experience sharing |
| 6 | Subjective norms | Aerobic activity such as brisk walking, running, or cycling | Activity tracking, peer discussion, and encouragement |
| 7 | Subjective norms | Strength exercises using app/video guidance, such as planks, push-ups, and abdominal exercises | Guided strength exercise and peer support |
| 8 | Subjective norms | Participation in team-based competitive activities | Team competition and social interaction |
| 9 | Perceived behavioral control | Exploration of exercise modalities according to personal preference and ability, such as rope skipping, combat exercise, tai chi, or dance | Exercise choice and autonomy |
| 10 | Perceived behavioral control | Achievement of an 8000-step goal with app-based monitoring | Goal setting and self-monitoring |
| 11 | Perceived behavioral control | Development and implementation of an exercise plan based on the participant’s own circumstances | Exercise planning and self-management |
| 12 | Perceived behavioral control | Creation of or participation in an app-based exercise group, including interaction and experience sharing | Social support and group engagement |

The intervention was delivered over 12 weeks, with three scheduled activity sessions per week. Weekly tasks were posted in advance through WeChat. Participants submitted app-generated screenshots for each scheduled intervention session, and submissions were reviewed by two researchers. Missing scheduled submissions were followed up on the same day. Moderate-intensity exercise sessions generally targeted RPE 12–14.

Table S2. Pilot CFA Standardized Factor Loadings, Average Variance Extracted, and Composite Reliability of the Retained Measurement Dimensions

| Construct | Dimension | Item | Standardized factor loading | AVE ^a^ | CR ^b^ |
| --- | --- | --- | --- | --- | --- |
| Sports app involvement | Attractiveness | A1 | 0.735 | 0.582 | 0.847 |
|  |  | A2 | 0.763 |  |  |
|  |  | A3 | 0.808 |  |  |
|  |  | A4 | 0.742 |  |  |
|  | Self-expression | A5 | 0.829 | 0.617 | 0.828 |
|  |  | A6 | 0.722 |  |  |
|  |  | A7 | 0.801 |  |  |
|  | Life centrality | A8 | 0.774 | 0.552 | 0.786 |
|  |  | A9 | 0.680 |  |  |
|  |  | A10 | 0.770 |  |  |
| Digitalization | Monitoring | B1 | 0.698 | 0.508 | 0.804 |
|  |  | B2 | 0.710 |  |  |
|  |  | B3 | 0.750 |  |  |
|  |  | B4 | 0.689 |  |  |
|  | Feedback | B5 | 0.662 | 0.522 | 0.813 |
|  |  | B6 | 0.656 |  |  |
|  |  | B7 | 0.778 |  |  |
|  |  | B8 | 0.783 |  |  |
| Gamification | Sense of mastery | C1 | 0.773 | 0.536 | 0.775 |
|  |  | C2 | 0.649 |  |  |
|  |  | C3 | 0.767 |  |  |
|  | Interaction fluency | C4 | 0.716 | 0.509 | 0.757 |
|  |  | C5 | 0.747 |  |  |
|  |  | C6 | 0.676 |  |  |
|  | Intrinsic motivation | C7 | 0.767 | 0.519 | 0.764 |
|  |  | C8 | 0.663 |  |  |
|  |  | C9 | 0.728 |  |  |
| Core literacy in physical education and health | Motor competence | D1 | 0.675 | 0.510 | 0.892 |
|  |  | D2 | 0.767 |  |  |
|  |  | D3 | 0.769 |  |  |
|  |  | D4 | 0.757 |  |  |
|  |  | D5 | 0.735 |  |  |
|  |  | D6 | 0.659 |  |  |
|  |  | D7 | 0.645 |  |  |
|  |  | D8 | 0.692 |  |  |
|  | Health behaviors | D10 | 0.685 | 0.563 | 0.934 |
|  |  | D11 | 0.730 |  |  |
|  |  | D12 | 0.763 |  |  |
|  |  | D13 | 0.762 |  |  |
|  |  | D15 | 0.789 |  |  |
|  |  | D16 | 0.737 |  |  |
|  |  | D17 | 0.809 |  |  |
|  |  | D18 | 0.788 |  |  |
|  |  | D19 | 0.604 |  |  |
|  |  | D20 | 0.747 |  |  |
|  |  | D21 | 0.813 |  |  |
|  | Sportsmanship | D22 | 0.765 | 0.568 | 0.901 |
|  |  | D23 | 0.604 |  |  |
|  |  | D24 | 0.828 |  |  |
|  |  | D25 | 0.755 |  |  |
|  |  | D26 | 0.834 |  |  |
|  |  | D27 | 0.661 |  |  |
|  |  | D28 | 0.800 |  |  |

^a^ AVE=average variance extracted.

^b^ CR=composite reliability.

Results are based on the pilot CFA subsample (n=96). AVE and CR are reported at the dimension level.

Table S3. Sources and Adaptation of the Study 2 Measurement Instruments

| Construct | Source basis | Adaptation in this study | Final structure |
| --- | --- | --- | --- |
| Sports app involvement | McIntyre and Pigram leisure involvement framework | Items contextualized to sports app use | 10 items: attractiveness (4), self-expression (3), life centrality (3) |
| Digitalization | Monitoring and feedback measures reported by Nelson et al. | Smart-wristband wording contextualized to sports apps | 8 items: monitoring (4), feedback (4) |
| Gamification | SDT and prior game-experience literature | Context-specific items developed and refined through literature review and expert consultation | 9 items: sense of mastery (3), interaction fluency (3), intrinsic motivation (3) |
| Core literacy in physical education and health | Chinese curriculum framework and earlier Chinese university-student instruments | Initial 30-item study-specific scale; 4 items removed after pilot item analysis | 26 items: motor competence (8), health behaviors (11), sportsmanship (7) |

The gamification and core-literacy instruments were developed or refined for the present study rather than directly adopted without modification from a single previously validated instrument. Detailed pilot psychometric results are reported in Table S2.

Table S4. Characteristics of the Study 1 Complete-Case Sample by Group

| Characteristic | PE ^a^ (*n*=28) | PC ^b^ (*n*=25) | UE ^c^ (*n*=25) | UC ^d^ (*n*=29) | Overall (*N*=107) |
| --- | --- | --- | --- | --- | --- |
| Male, n (%) | 12 (43%) | 15 (60%) | 11 (44%) | 11 (38%) | 49 (46%) |
| Age, years | 20.57 ± 1.37 | 20.32 ± 1.41 | 20.80 ± 1.91 | 20.55 ± 1.66 | 20.56 ± 1.59 |
| Height, cm | 172.36 ± 9.44 | 172.88 ± 8.27 | 172.00 ± 8.95 | 167.69 ± 9.48 | 171.13 ± 9.20 |
| Weight, kg | 63.75 ± 14.36 | 62.16 ± 12.93 | 62.60 ± 13.49 | 59.90 ± 13.26 | 62.07 ± 13.42 |

^a^ PE pretested experimental group.

^b^ PC pretested control group.

^c^ UE unpretested experimental group.

^d^ UC unpretested control group.

Characteristics are reported for participants with available postintervention outcome data.

Table S5. Baseline Comparison Between Completers and Noncompleters in the Pretested Groups

| Baseline characteristic | Completers (n=53) | Noncompleters (n=7) | *P* value |
| --- | --- | --- | --- |
| Male, n (%) | 27 (51%) | 4 (57%) | > .99 |
| Age, years | 20.36 ± 1.72 | 20.29 ± 0.49 | .81 |
| Height, cm | 172.30 ± 9.06 | 171.71 ± 6.40 | .83 |
| Weight, kg | 63.28 ± 13.49 | 66.29 ± 12.13 | .56 |
| BMI | 21.15 ± 3.27 | 22.31 ± 2.36 | .27 |
| Motor competence | 28.08 ± 3.94 | 29.43 ± 1.51 | .10 |
| Health behaviors | 40.30 ± 4.13 | 41.14 ± 3.39 | .56 |
| Sportsmanship | 27.94 ± 2.37 | 28.71 ± 1.60 | .29 |
| Total core literacy score | 96.32 ± 7.35 | 99.29 ± 3.15 | .07 |

Values are mean±SD unless otherwise indicated. Continuous variables were compared using Welch independent-samples t tests; sex was compared using Fisher exact test. Baseline core literacy measurements were administered only to participants in the pretested experimental and pretested control groups as specified by the Solomon four-group design.

Table S6. Reference-Based Attrition Sensitivity Analysis for Study 1

| Outcome | Median F | 2.5th-97.5th percentile | Median ηp^²^ | Replications with P<.05, n (%) |
| --- | --- | --- | --- | --- |
| Motor competence | 27.60 | 19.33-37.12 | 0.192 | 10,000 (100%) |
| Health behaviors | 17.94 | 10.87-25.55 | 0.134 | 10,000 (100%) |
| Sportsmanship | 24.52 | 16.79-33.46 | 0.175 | 10,000 (100%) |

Results are based on 10,000 reference-based resampling replications. Percentile ranges represent the empirical 2.5th and 97.5th percentiles across replications and are not conventional confidence intervals.

Table S7. Detailed Exploratory Tukey HSD Pairwise Comparisons Among Solomon Design Observations

| Dependent Variable | Group I | Group J | Mean Difference (I-J) | Standard Error | *P* value | 95% Confidence Interval | |
| --- | --- | --- | --- | --- | --- | --- | --- |
|  |  |  |  |  |  | Lower limit | Upper limit |
| Motor competence | O1 | O2 | -6.607 | 0.982 | <.001 | -9.443 | -3.772 |
|  | O1 | O3 | 1.733 | 1.012 | .53 | -1.186 | 4.652 |
|  | O2 | O4 | 4.100 | 1.012 | .001 | 1.181 | 7.019 |
|  | O2 | O5 | 3.340 | 1.012 | .02 | 0.421 | 6.259 |
|  | O3 | O4 | -4.240 | 1.040 | .001 | -7.241 | -1.239 |
|  | O3 | O6 | -1.047 | 1.003 | .90 | -3.942 | 1.848 |
|  | O4 | O6 | 3.193 | 1.003 | .02 | 0.298 | 6.088 |
|  | O5 | O6 | 3.953 | 1.003 | .002 | 1.058 | 6.848 |
| Health behaviors | O1 | O2 | -7.393 | 1.180 | <.001 | -10.799 | -3.986 |
|  | O1 | O3 | 0.571 | 1.215 | >.99 | -2.936 | 4.079 |
|  | O2 | O4 | 4.004 | 1.215 | .02 | 0.497 | 7.512 |
|  | O2 | O5 | 3.804 | 1.215 | .03 | 0.297 | 7.312 |
|  | O3 | O4 | -3.960 | 1.249 | .02 | -7.565 | -0.355 |
|  | O3 | O6 | 0.000 | 1.205 | >.99 | -3.479 | 3.479 |
|  | O4 | O6 | 3.960 | 1.205 | .02 | 0.481 | 7.439 |
|  | O5 | O6 | 4.160 | 1.205 | .009 | 0.681 | 7.639 |
| Sportsmanship | O1 | O2 | -3.750 | 0.669 | <.001 | -5.681 | -1.819 |
|  | O1 | O3 | 0.120 | 0.689 | >.99 | -1.868 | 2.108 |
|  | O2 | O4 | 2.590 | 0.689 | .003 | 0.602 | 4.578 |
|  | O2 | O5 | 0.790 | 0.689 | .86 | -1.198 | 2.778 |
|  | O3 | O4 | -1.280 | 0.708 | .46 | -3.323 | 0.763 |
|  | O3 | O6 | -0.223 | 0.683 | >.99 | -2.195 | 1.748 |
|  | O4 | O6 | 1.057 | 0.683 | .64 | -0.915 | 3.028 |
|  | O5 | O6 | 2.857 | 0.683 | <.001 | 0.885 | 4.828 |

These pairwise comparisons are exploratory and descriptive because the Pretest × Intervention interactions were not statistically significant in the primary factorial analysis.

Table S8. Characteristics of the Study 2 Cross-Sectional Sample

| Characteristic | Overall (N=790) |
| --- | --- |
| Male, n (%) | 409 (51.8%) |
| Female, n (%) | 381 (48.2%) |
| Age, years | 20.33 ± 1.78 |
| Height, cm | 171.95 ± 10.11 |
| Weight, kg | 63.67 ± 12.85 |
| BMI | 21.36 ± 2.85 |
| Qufu Normal University, n (%) | 117 (14.8%) |
| Shandong Jiaotong University, n (%) | 115 (14.6%) |
| Shandong University, n (%) | 105 (13.3%) |
| Jining University, n (%) | 104 (13.2%) |
| Dezhou University, n (%) | 101 (12.8%) |
| Qingdao Agricultural University, n (%) | 97 (12.3%) |
| Ludong University, n (%) | 90 (11.4%) |
| Liaocheng University, n (%) | 61 (7.7%) |

Table S9. University-Level Clustering and Sensitivity Analysis

Panel A. University-Level Clustering

| Construct | ICC | Design Effect |
| --- | --- | --- |
| Sports app involvement | 0.010 | 1.99 |
| Digitalization | 0.002 | 1.18 |
| Gamification | ≈0 | 1.00 |
| Core literacy | ≈0 | 1.00 |

Panel B. Covariate Sensitivity Analysis

| Path | Age- and Sex-Adjusted β | Expanded Covariate-Adjusted β |
| --- | --- | --- |
| Sports app involvement → Core literacy | .155 | .151 |
| Sports app involvement → Digitalization | .538 | .545 |
| Digitalization → Core literacy | .300 | .310 |
| Sports app involvement → Gamification | .273 | .270 |
| Gamification → Core literacy | .152 | .145 |
| Digitalization → Gamification | .383 | .391 |

Panel C. University-Clustered Robust Sensitivity Analysis

| Path | β | Cluster-Robust SE | P value |
| --- | --- | --- | --- |
| Sports app involvement → Core literacy | 0.155 | 0.039 | .006 |
| Sports app involvement → Digitalization | 0.538 | 0.030 | <.001 |
| Digitalization → Core literacy | 0.300 | 0.039 | <.001 |
| Sports app involvement → Gamification | 0.273 | 0.030 | <.001 |
| Gamification → Core literacy | 0.152 | 0.062 | .04 |
| Digitalization → Gamification | 0.383 | 0.032 | <.001 |

ICCs were estimated using construct composite scores. Panel B presents a secondary sensitivity path analysis based on standardized composite scores rather than the latent-variable SEM used for the primary analysis. The expanded model additionally included BMI and 7 university indicator variables while retaining age and sex as covariates. All six focal paths in Panel B remained statistically significant at *P*<.001. Absolute changes in standardized coefficients between the age- and sex-adjusted and expanded composite-score models did not exceed 0.010. Panel C reports university-clustered robust standard errors for the age- and sex-adjusted composite-score models, with a finite-sample correction and t-based inference using 7 degrees of freedom (8 university clusters). β represents the standardized coefficient, and SE is reported on the same scale. These results were interpreted as sensitivity evidence given the small number of university clusters.

Table S10. Heterotrait-Monotrait Ratios Among the Study 2 Constructs

|  | 1 | 2 | 3 | 4 |
| --- | --- | --- | --- | --- |
| 1 Sports app involvement | — |  |  |  |
| 2 Digitalization | 0.621 | — |  |  |
| 3 Gamification | 0.559 | 0.638 | — |  |
| 4 Core literacy | 0.426 | 0.527 | 0.448 | — |

All HTMT values were below the conservative 0.85 criterion.

Table S11. Standardized Factor Loadings, Composite Reliability, and Average Variance Extracted in the Formal Study 2 Sample

| Construct | Dimension-Level Indicator | Standardized Loading | CR | AVE |
| --- | --- | --- | --- | --- |
| Sports app involvement | Attractiveness | 0.831 | 0.841 | 0.639 |
|  | Self-expression | 0.787 |  |  |
|  | Life centrality | 0.778 |  |  |
| Digitalization | Monitoring | 0.887 | 0.808 | 0.680 |
|  | Feedback | 0.757 |  |  |
| Gamification | Sense of mastery | 0.703 | 0.770 | 0.527 |
|  | Interaction fluency | 0.707 |  |  |
|  | Intrinsic motivation | 0.767 |  |  |
| Core literacy in physical education and health | Motor competence | 0.804 | 0.865 | 0.683 |
|  | Health behaviors | 0.906 |  |  |
|  | Sportsmanship | 0.763 |  |  |

Results are based on the formal Study 2 sample (N=790). Standardized loadings are reported for the 11 dimension-level indicators used in the four-factor measurement model. CR=composite reliability; AVE=average variance extracted. All CR values exceeded 0.70 and all AVE values exceeded 0.50. No item deletion or measurement-model reoptimization was performed using the formal Study 2 sample.
